# Early Versus Delayed Oxytocin Augmentation in Nulliparous Women with Spontaneous Dysfunctional Labour: A Systematic Review and Meta-Analysis

**DOI:** 10.3390/healthcare14183000

**Published:** 2026-09-14

**Authors:** Ilenia Mappa, Martina Derme, Francesco Maria Maruotti, Francesco D’Antonio, Giuseppe Rizzo

**Affiliations:** 1 Department of Obstetrics and Gynecology, Sapienza University of Rome, 00185 Rome, Italy; ilenia.mappa@uniroma1.it (I.M.); martina.derme@uniroma1.it (M.D.); 2 Department of Obstetrics and Gynecology, University of Naples Federico II, 80131 Naples, Italy; francescomaria.maruotti@unina.it; 3 Center for Fetal Care and High-Risk Pregnancy, Department of Obstetrics and Gynecology, University “G. d’Annunzio” of Chieti-Pescara, 66100 Chieti, Italy; francesco.dantonio@unich.it

**Keywords:** oxytocin, labour augmentation, dysfunctional labour, nulliparity, caesarean section, uterine hyperstimulation, meta-analysis

## Abstract

**Background**: The timing of oxytocin for slow labour progress rests on custom rather than evidence, and existing syntheses pool clinically distinct populations. **Objectives**: We restricted this systematic review to nulliparous women in established active-phase labour with a trial-specified diagnosis of slow progress, comparing early versus delayed or withheld intravenous oxytocin. **Methods**: Cochrane review CD007123 was updated by searching MEDLINE, Embase and CENTRAL; counts were recomputed from primary reports. **Results**: Three trials (1102 women) were eligible. Early oxytocin did not alter caesarean delivery (three trials, 1102 women; risk ratio [RR] 0.86, 95% confidence interval [CI] 0.63–1.16), an interval excluding neither benefit nor harm. Uterine hyperstimulation, defined differently in the two contributing trials, was more frequent with early oxytocin (two trials, 472 women; RR 2.51, 95% CI 1.04–6.06), but 92% of the weight came from one trial and the association did not survive Hartung–Knapp or leave-one-out analysis. No difference was demonstrated for instrumental or spontaneous delivery, Apgar < 7, neonatal admission or haemorrhage. Labour was shorter by two hours (two trials, 1042 women; mean difference −2.17 h, 95% CI −3.54 to −0.80), with substantial heterogeneity. Certainty was low or very low. **Conclusions**: Early oxytocin may shorten labour; its effects on caesarean delivery and other outcomes remain uncertain.

## 1. Introduction

Oxytocin is among the most frequently administered drugs on labour wards, yet the interval between the diagnosis of slow progress in the first stage and the start of the infusion is governed in most units by local custom rather than by evidence. The rationale inherited from the active management of labour maintains that prompt augmentation restores normal progress and averts caesarean delivery [1]. The World Health Organization takes the opposite view, recommending against augmentation before delay has been confirmed, on the grounds that unnecessary exposure risks uterine hyperstimulation and foetal compromise [2]. This caution is reinforced by contemporary cohort data showing that first-stage progression is frequently slower than the historical benchmark of 1 cm/h, so that a rigid threshold risks labelling normal labour as dysfunctional [3]; the 2018 WHO intrapartum-care recommendations accordingly place the onset of the active first stage at 5 cm and discourage routine early intervention [4], a change operationalised in the WHO Labour Care Guide [5].

The randomised evidence has not resolved this tension, and the existing syntheses are structurally ill-suited to doing so. The parent Cochrane review of this question [6] pooled two comparisons—oxytocin versus placebo, and early versus delayed oxytocin—across populations that are not clinically interchangeable: nulliparous women were combined with parous women, and augmentation of established spontaneous labour was combined with augmentation after amniotomy. The nullipara in spontaneous labour—a woman in whom dysfunctional labour is common and for whom the caesarean decision is most often made—has rarely been analysed on her own.

We therefore undertook a systematic review restricted to a single, clinically coherent population: nulliparous women in established spontaneous labour with a prespecified diagnosis of slow progress. Our aim was to determine whether the timing of oxytocin, rather than its use versus non-use, alters the mode of delivery, intrapartum safety, or the duration of labour.

## 2. Materials and Methods

The review was reported in accordance with the PRISMA 2020 statement [7] and conducted to a protocol prepared per PRISMA-P [8] (Supplementary Material Table S1). It was prospectively registered with PROSPERO (registration number CRD420261451210). The parity- and comparison-restricted question, the outcome set, and the risk-of-bias and certainty frameworks were prespecified.

### 2.1. Eligibility Criteria

We included randomised controlled trials of nulliparous women (no previous birth ≥ 24 weeks) with a singleton cephalic pregnancy at term, in established spontaneous labour, in whom slow progress had been diagnosed by the trial’s own prespecified criteria, randomised to early versus delayed or withheld intravenous oxytocin. Eligibility was restricted to the established active first stage of labour: trials of prolonged latent phase and trials of general active-management-of-labour packages in which augmentation was not the randomised variable were not eligible. No single common dilatation threshold was imposed; the diagnostic criterion for slow progress was the one prespecified by each trial, and these trial-specific thresholds are reported in Table 1 so that the population actually studied is transparent. The randomised variable had to be the timing of oxytocin; a co-intervention applied identically to both arms (for example, amniotomy in all women with intact membranes) did not disqualify a trial. We excluded trials of induced labour, trials of mixed parity in which nulliparous data could not be separated, trials whose comparator was non-pharmacological, and trials whose population was not restricted to confirmed slow progress. A complete list of full-text exclusions with study-specific reasons is provided in Table S4.

### 2.2. Information Sources and Search

The trials included in Cochrane review CD007123 [6] (search to February 2013) were used as the seed set. Because that search predates more than a decade of the subsequent literature, we updated it by searching MEDLINE (via PubMed), Embase and the Cochrane Central Register of Controlled Trials (CENTRAL) from inception to 16 July 2026, with no language or publication-status restriction, supplemented by forward citation tracking of the included trials and by screening the reference lists of the included trials and of relevant guidelines. The complete, reproducible search strategy for each database—full search strings, date limits and any filters applied—is reported in the Supplementary Materials (Table S2), together with the completed PRISMA 2020 checklist (Table S3). CINAHL and Web of Science were not searched independently; this is acknowledged as a limitation.

### 2.3. Study Selection and Data Extraction

Two reviewers independently screened records and extracted data onto a piloted form; disagreements were resolved by a third reviewer. For every trial, per-arm event counts and denominators were taken from the primary report, and the internal consistency of the data was checked by confirming that the modes of delivery summed to each arm’s randomised denominator. Pooled estimates were recomputed de novo from source counts and were not transcribed from any previous synthesis.

### 2.4. Risk of Bias and Certainty of Evidence

Risk of bias was assessed with the Cochrane RoB 2 tool [12], per outcome, independently by two reviewers. Certainty of evidence was rated with GRADE [13] and, for the primary outcome, imprecision was judged against an optimal information size.

### 2.5. Synthesis

Dichotomous outcomes were expressed as risk ratios (RR) with 95% confidence intervals (CI) and pooled by the DerSimonian–Laird random-effects model [14] and prespecified as the primary model because the duration of delay and the criteria for initiating oxytocin in the control arm were not uniform across trials. Continuous outcomes were pooled as mean differences (MD). Heterogeneity was quantified with I^2^ and τ^2^. A continuity correction of 0.5 was applied only to cells with zero events. Publication bias was prespecified to be assessed by funnel plot and Egger’s test only for outcomes informed by ten or more trials; because no outcome met this threshold, formal assessment was not performed. Postpartum haemorrhage was analysed by the definition used in each trial rather than by pooling dissimilar thresholds. We recognise that the DerSimonian–Laird estimator, although prespecified, is not ideal here: only two or three trials contributed to each outcome, and with so few studies, the between-study variance is estimated imprecisely and DerSimonian–Laird intervals can be too narrow. We therefore added a post hoc sensitivity analysis in which τ^2^ was re-estimated by restricted maximum likelihood (REML) and the confidence interval was derived using the Hartung–Knapp adjustment for the two dichotomous outcomes to which more than one trial contributed. For the same reason, an observed I^2^ of 0% across two or three trials should not be interpreted as evidence of true homogeneity: with so few studies, I^2^ carries very wide uncertainty and has little power to detect real between-trial variation. Because a random-effects model is hard to justify when τ^2^ cannot be estimated, we additionally report a fixed-effect (inverse-variance) estimate and a leave-one-out analysis for both outcomes, and we interpret the individual trial results narratively alongside any pooled figure. All analyses were performed using StatsDirect Statistical Software Version 4.04 (StatsDirect Ltd., Cambridge, UK), with the sensitivity analyses computed in R (metafor version 2026-06-21).

## 3. Results

### 3.1. Study Selection

**Figure 1:**
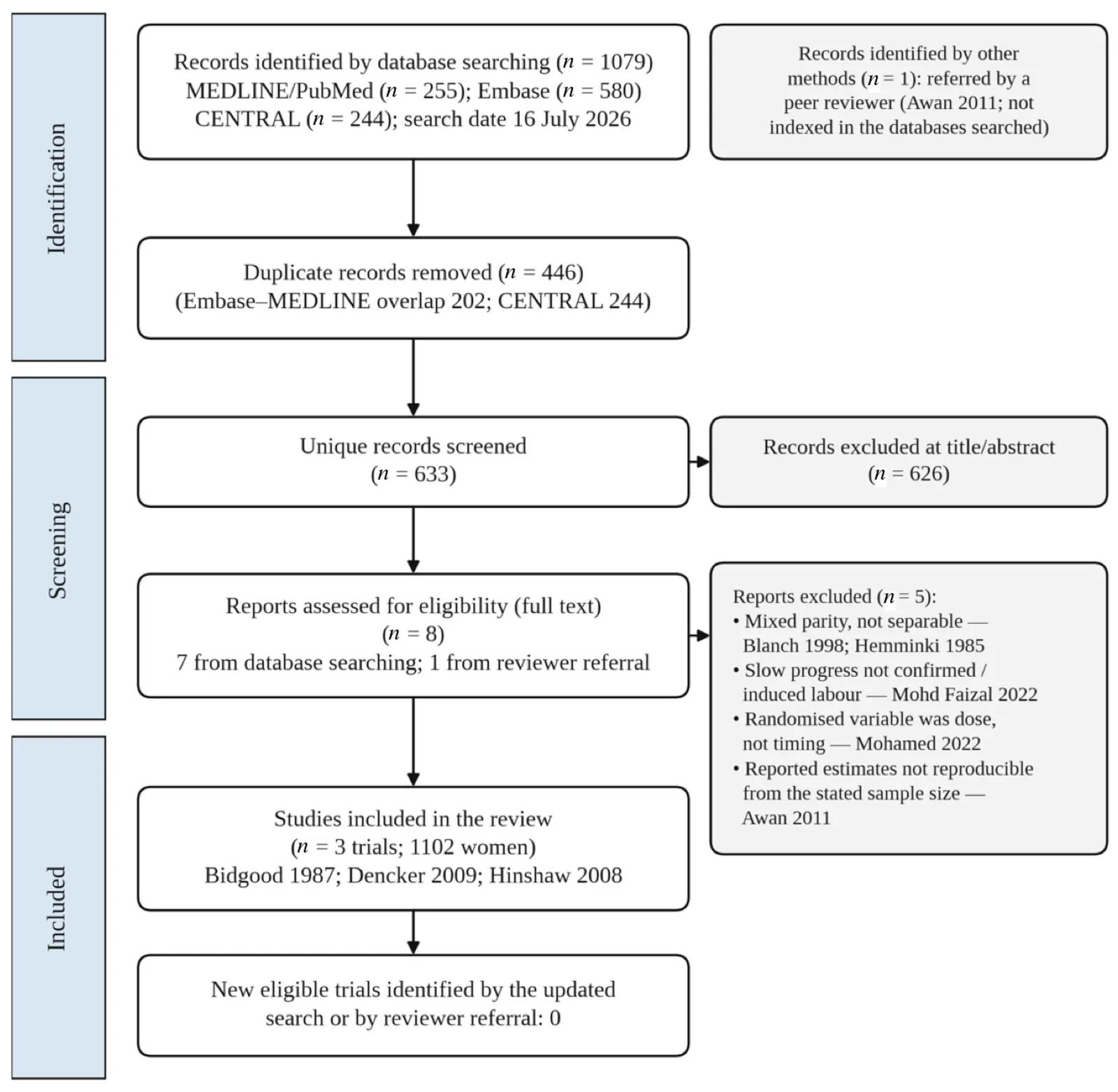
Study selection (PRISMA flow). Counts reflect the updated search of MEDLINE, Embase and CENTRAL to 16 July 2026, together with one report identified through peer-reviewer referral [9,10,11,15,16,17,18,20,22].

The updated search identified 1079 records (255 from MEDLINE/PubMed, 580 from Embase and 244 from CENTRAL); after the removal of 446 duplicates, 633 unique records were screened. Of the five early-versus-delayed trials in the parent review, three enrolled nulliparous women exclusively and these were included (Bidgood 1987 [9,15], Dencker 2009 [10], Hinshaw 2008 [11]; 1102 women). Two were excluded because they enrolled a mixed-parity population that could not be separated—Blanch 1998 [16] combined nulliparous and parous women in a single randomised cohort, and Hemminki 1985 [17] reported outcomes for a mixed-parity sample without a nulliparous subgroup, so neither could satisfy the prespecified parity restriction. The updated search identified two candidates published after the parent review; both were excluded from the full text. Mohd Faizal 2022 [18] was excluded because its population was not restricted to confirmed slow progress (women were enrolled at 4 cm dilatation) and included a substantial proportion of induced labours (16.5%) that could not be separated in the outcome data; that trial belongs instead to the literature on early amniotomy with oxytocin [19]. Mohamed 2022 [20] was excluded because its randomised variable was oxytocin dose (high versus low), not timing, in an obese parous population; it belongs to the high-versus-low-dose literature [21]. One further report, Awan 2011 [22], was brought to our attention during peer review. It carries no DOI and is not indexed in MEDLINE, Embase or CENTRAL, and it was therefore reachable neither by the database search nor by forward citation tracking, both of which depend on a record existing in an indexing service or with a registration agency. No search strategy confined to those sources could have retrieved it. At first glance, it meets the eligibility criteria—nulliparous women at term in spontaneous labour, randomised after a 2 h arrest in cervical dilatation to oxytocin within 20 min (*n* = 157) or to postponement for 3 h (*n* = 158). We obtained the full report and examined it against the eligibility criteria and for internal consistency. The confidence intervals it reports are not reproducible from the sample size it states: for the arm sizes given, the interval around the reported caesarean odds ratio of 0.8 would be approximately 0.39–1.71 rather than the 0.5–1.4 reported, and the interval around the reported instrumental-delivery odds ratio of 1.5 would be approximately 0.81–2.86 rather than 0.97–2.4; the reported intervals correspond instead to a sample of roughly twice the stated size. No per-arm event counts are given from which the discrepancy could be resolved, and it cannot be resolved from any other part of the published report. We therefore could not verify that the reported estimates derive from the sample described, could not extract data we were able to check, and excluded the report on that ground. No trial was added to the review by this route. The selection process is shown in Figure 1.

### 3.2. Characteristics and Risk of Bias

**Figure 2:**
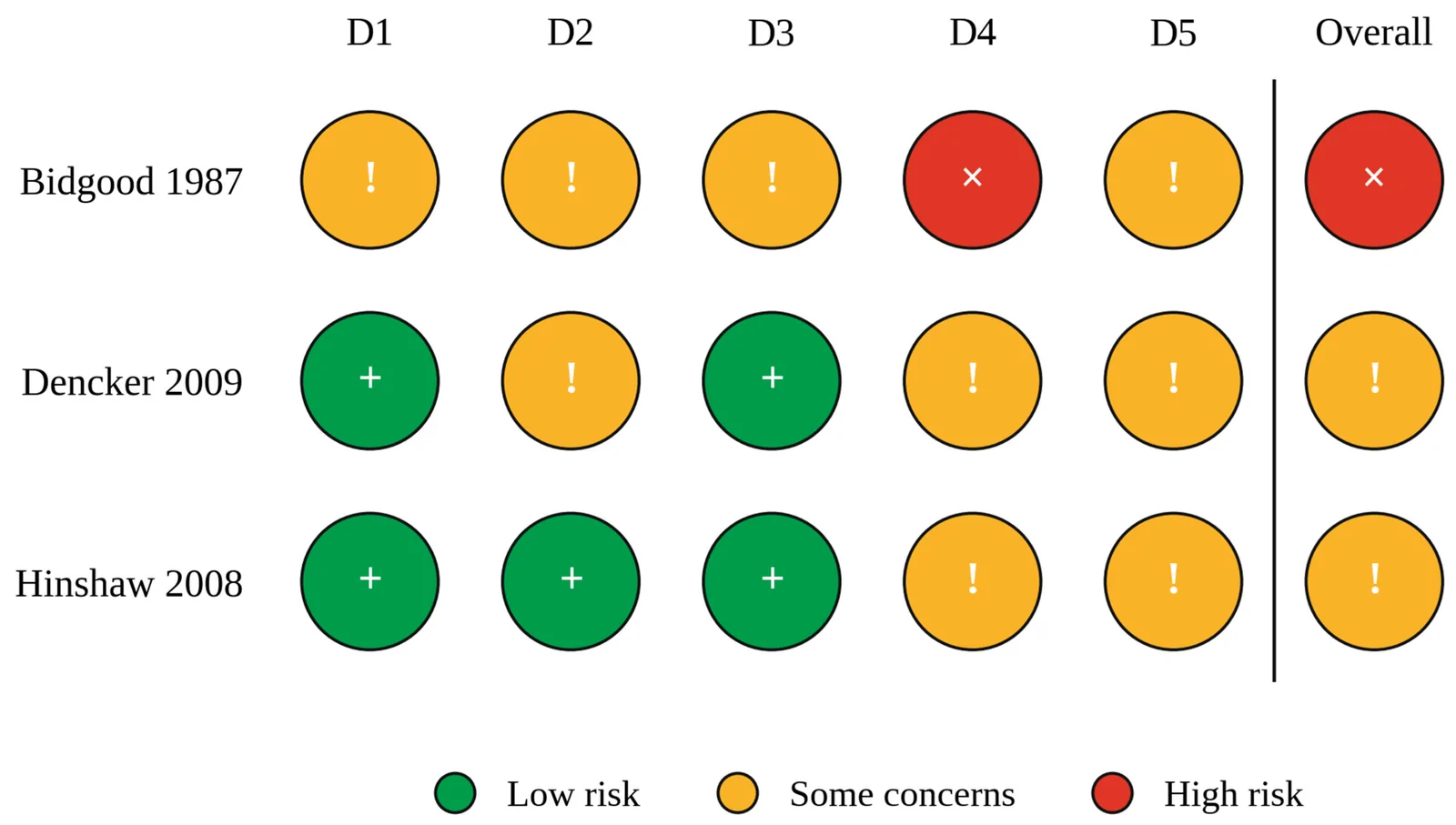
Risk of bias in the included trials (RoB 2), by domain and overall. D1, randomisation process; D2, deviations from intended interventions; D3, missing outcome data; D4, measurement of the outcome; D5, selection of the reported result [9,10,11,15].

The included trials are summarised in Table 1. All enrolled nulliparous women in spontaneous labour with a prespecified diagnosis of slow progress, and all performed amniotomy in women with intact membranes before randomisation. None used a placebo or blinded participants or clinicians; all three were therefore open-label. The delayed arm was not uniform: the withheld interval ranged from 3 to 8 h, and in the largest trial, 13% of the delayed group ultimately received no oxytocin while a further ~20% received it earlier than allocated. The delayed arm therefore does not represent a single therapeutic strategy: what was compared was early augmentation against a heterogeneous set of expectant or deferred-augmentation strategies, differing both in the intended length of delay and in the proportion of women eventually treated. Risk of bias is summarised in Table 2 and displayed in Figure 2; every trial raised at least some concerns, principally from the absence of blinding of clinician-driven outcomes and from the pre-registration reporting environment. Because no trial was blinded, and because the decision to proceed to caesarean or instrumental delivery is taken by the attending clinician in real time, knowledge of allocation could have acted directly on these outcomes rather than only on their ascertainment; measurement (decision) bias is therefore the dominant bias concern for the mode-of-delivery outcomes rather than a peripheral one. Risk of bias was judged per outcome and the judgements are not uniform across outcomes: for caesarean and instrumental delivery, all three trials were rated high or some concerns in the measurement domain, whereas for the objectively recorded neonatal outcomes (Apgar, neonatal intensive care admission), the same domain was rated low. For uterine hyperstimulation, the measurement domain was rated high in both contributing trials, because the outcome was neither blinded nor defined identically. Table 2 reports the trial-level summary; the full outcome-specific RoB 2 judgements with supporting rationales are given as Table S5.

### 3.3. Caesarean Delivery (Primary Outcome)

**Figure 3:**
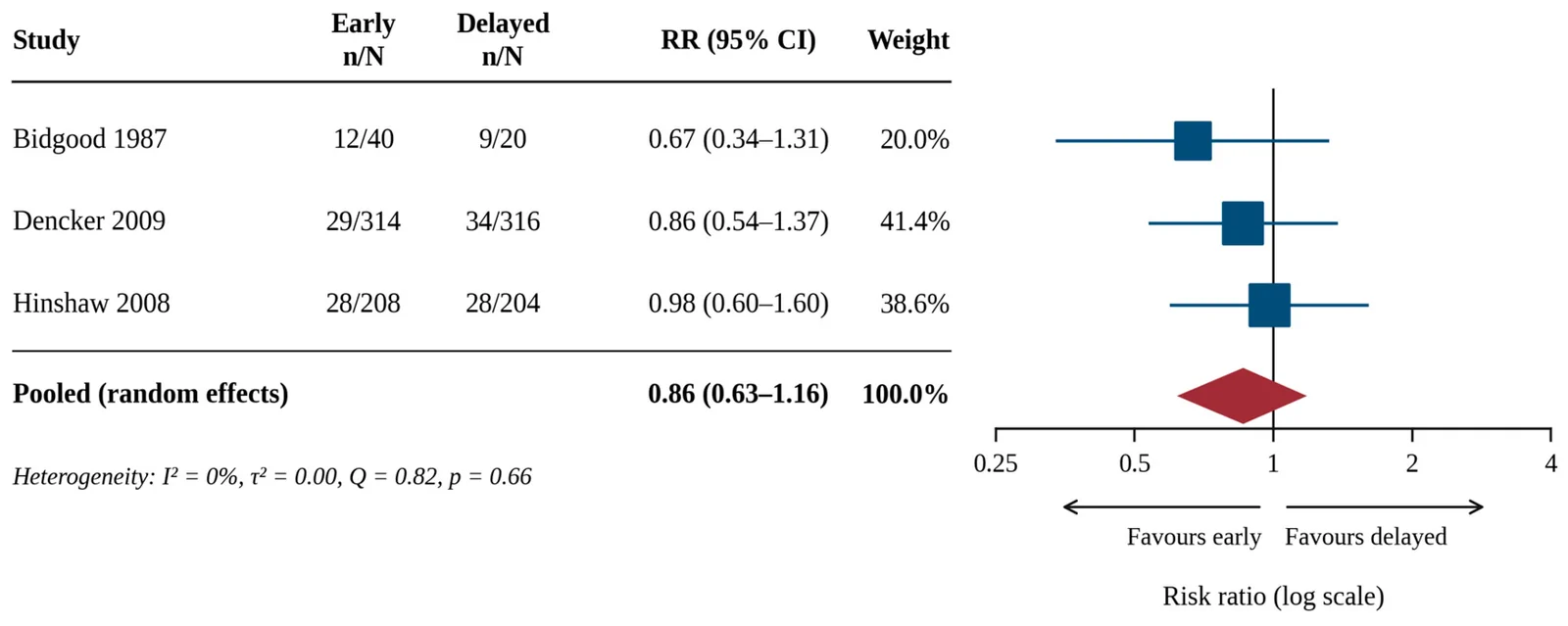
Caesarean section (primary outcome) [9,10,11,15].

Early oxytocin did not alter the risk of caesarean delivery (RR 0.86, 95% CI 0.63–1.16; I^2^ = 0%; three trials, 1102 women; Figure 3). The confidence interval excludes a benefit or harm larger than approximately one-third but is compatible with the modest reductions that motivate the intervention. The available sample of 1102 women falls short of the optimal information size. Assuming the pooled control-arm caesarean rate observed across the delayed arms (71/540, 13.1%), a two-sided α of 0.05, 80% power and a clinically meaningful 30% relative risk reduction (13.1% to 9.2%), approximately 2000 women would be required, rising to approximately 2680 women at 90% power; the 1102 women available represent 55% and 41% of these totals, respectively. The primary comparison is therefore underpowered despite the null point estimate. The absence of a statistically significant difference does not establish equivalence: the interval remains compatible with a clinically important benefit and with clinically important harm.

### 3.4. Uterine Hyperstimulation (Trial-Specific Definitions)

**Figure 4:**
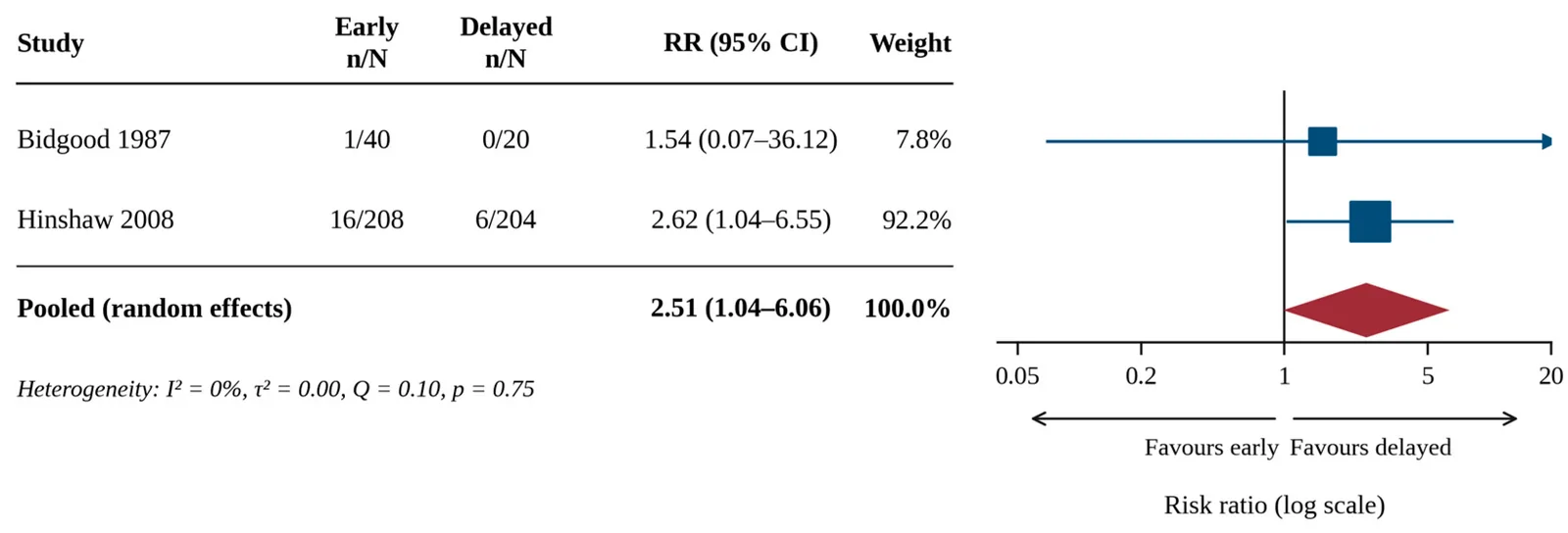
Uterine hyperstimulation as defined in each trial (qualified by fatal heart rate changes in Bidgood 1987 only [15]) [11,15].

The only outcome for which a difference was observed was uterine hyperstimulation, reported under definitions that were not equivalent across the two contributing trials: in Bidgood 1987 [15], the event was explicitly qualified by foetal heart rate change (a single case of hyperstimulation with persistent late decelerations), whereas Hinshaw 2008 [11] reported uterine hyperstimulation without stating that every event was accompanied by fatal heart rate abnormality. We have therefore relabelled this outcome according to the original trial definitions rather than as “hyperstimulation with foetal heart rate changes”. Because the equivalence of the two definitions cannot be established from the published reports, the pooled figure should be read as exploratory. Events were more frequent in the early-oxytocin arms (RR 2.51, 95% CI 1.04–6.06; I^2^ = 0%; two trials, 472 women; Figure 4). This is not corroboration across independent trials: Hinshaw 2008 [11] carries 92% of the weight and Bidgood 1987 [15] contributes a single event, so the pooled estimate is close to a restatement of the Hinshaw result. Leave-one-out analysis makes the dependency explicit—removing Hinshaw 2008 [11] leaves RR 1.54 (95% CI 0.07–36.12) from one event, and no meaningful pooled effect remains (Section 3.8)—and under REML with the Hartung–Knapp adjustment, the interval widens to 0.41–15.36 and no longer excludes unity. Taken with the sparse events and the non-equivalent definitions, this result is best described as an uncertain safety signal, largely dependent on a single trial, rather than as a demonstrated increase in risk.

### 3.5. Other Delivery, Neonatal and Maternal Outcomes

No difference was demonstrated between the arms for any other outcome. Instrumental vaginal delivery (RR 1.03, 95% CI 0.66–1.61; I^2^ = 69%) and spontaneous vaginal delivery (RR 1.04, 95% CI 0.88–1.25; I^2^ = 59%) were both null, with substantial heterogeneity reflecting effects in both directions. No difference was demonstrated for neonatal outcomes: 5 min Apgar < 7 (RR 1.02, 95% CI 0.43–2.40; I^2^ = 0%; three trials, 1102 women) and admission to neonatal intensive care (RR 1.03, 95% CI 0.64–1.66; I^2^ = 0%; two trials, 1042 women—Dencker 2009 and Hinshaw 2008 [10,11], the only trials reporting this outcome). Serious neonatal morbidity or perinatal death was reported by a single trial (one death in each arm, both plausibly unrelated to the intervention). The confidence intervals around these estimates are wide and remain compatible with clinically relevant differences in either direction; the absence of a demonstrated difference should not be read as evidence of equivalence or as excluding a neonatal effect.

Postpartum haemorrhage was reported under different definitions and was not pooled across dissimilar thresholds: major haemorrhage (>1000 mL) did not differ in the one trial that reported it (RR 0.58, 95% CI 0.28–1.20), and any haemorrhage (>500 mL) did not differ in the other (RR 0.89, 95% CI 0.61–1.30). During extraction, we identified a transcription error in the parent review, in which the delayed-arm haemorrhage count of one trial had been entered as 42 rather than the 45 reported in the primary paper (a value confirmed by that trial’s own odds ratio); correcting it left the outcome null and reduced the apparent heterogeneity.

### 3.6. Duration of Labour

The interval from randomisation to delivery was shorter with early oxytocin, by just over two hours (MD −2.17 h, 95% CI −3.54 to −0.80; two trials, 1042 women), but with high heterogeneity (I^2^ = 90%). The direction of this effect is consistent across trials; its magnitude is not estimated precisely.

### 3.7. Certainty of Evidence

GRADE ratings are shown in Table 3. Certainty was low for caesarean delivery (downgraded for risk of bias and imprecision), very low for hyperstimulation (risk of bias, imprecision and indirectness arising from the non-uniform outcome definition), and low for the remaining outcomes. The sensitivity analysis reported in Section 3.8 did not alter these ratings; it reinforces the very-low rating assigned to hyperstimulation. The reasons for each downgrade are as follows. Caesarean delivery: risk of bias (open-label, clinician-driven outcome) −1; imprecision (sample 41–55% of the optimal information size, interval compatible with benefit and harm) −1; no downgrade for inconsistency or indirectness; publication bias not assessable with three trials and not downgraded. Uterine hyperstimulation: risk of bias −1; indirectness (non-equivalent outcome definitions) −1; imprecision (472 women, sparse events, interval unstable under sensitivity analysis) −1. Remaining outcomes: risk of bias −1 and imprecision −1, with inconsistency additionally noted but not separately downgraded for instrumental and spontaneous delivery, where I^2^ was 69% and 59%.

### 3.8. Sensitivity Analysis

Re-estimating τ^2^ by restricted maximum likelihood did not change either pooled point estimate, because the observed between-trial variance was zero for both outcomes. Applying the Hartung–Knapp adjustment widened both intervals: caesarean delivery RR 0.86 (95% CI 0.56–1.32) and uterine hyperstimulation RR 2.51 (95% CI 0.41–15.36). The conclusion for caesarean delivery is unchanged. The hyperstimulation result, by contrast, is not robust to the choice of variance estimator and interval method: with only two contributing trials, the Hartung–Knapp interval is necessarily very wide, and the nominal statistical significance of the DerSimonian–Laird estimate should not be relied upon. The time-to-delivery outcome, informed by two trials with I^2^ = 90%, is subject to the same instability, and its confidence interval is likely to be optimistically narrow. Because τ^2^ was estimated as zero, the fixed-effect (inverse-variance) estimates coincide with the random-effects estimates to two decimal places for both outcomes: caesarean delivery RR 0.86 (95% CI 0.63–1.16) and uterine hyperstimulation RR 2.51 (95% CI 1.04–6.06); the Mantel–Haenszel fixed-effect estimate for caesarean delivery was RR 0.85. Leave-one-out analysis is stable for caesarean delivery (omitting Bidgood 1987, RR 0.92, 95% CI 0.65–1.28; omitting Dencker 2009, RR 0.86, 95% CI 0.58–1.28; omitting Hinshaw 2008, RR 0.79, 95% CI 0.54–1.16) [9,10,11,15]. For hyperstimulation, it is not: omitting Bidgood 1987 leaves the Hinshaw estimate essentially unchanged (RR 2.62, 95% CI 1.04–6.55), whereas omitting Hinshaw 2008 leaves a single event in Bidgood 1987 (RR 1.54, 95% CI 0.07–36.12) and no informative pooled effect [9,10,11,15]. The safety signal therefore rests on one trial and is not corroborated by a second.

## 4. Discussion

In a nulliparous woman with spontaneous dysfunctional labour, early oxytocin shortens labour but has not been shown to make delivery safer. It did not reduce caesarean or instrumental delivery, did not improve neonatal outcomes, and was associated with a possible increase in uterine hyperstimulation, an outcome the two contributing trials did not define identically. The intervention buys time, not a safer birth. This is a coherent message for counselling: the woman may reasonably choose a shorter labour, but she should not be told that earlier oxytocin lowers her chance of caesarean. What these data do not establish is the converse—that delaying augmentation is itself superior. The findings of this meta-analysis and the recommendations that follow from contemporary guidelines are separate matters, and we keep them separate below. A further point of scope deserves emphasis: all three trials randomised women after a trial-specific diagnosis of slow progress had already been made, and the comparison was between immediate augmentation and delays of differing length. The review therefore addresses when to start oxytocin once delay has been diagnosed; it does not test whether oxytocin should be withheld until delay is first confirmed, and it says nothing about the prolonged latent phase.

By restricting to the nullipara in spontaneous labour with confirmed slow progress, this review isolates the population in which the clinical question actually arises, and it recomputes every estimate from the primary reports rather than inheriting them. Two consequences of that discipline are worth stating. First, an updated search of three databases identified no new eligible trial: the principal post-2013 candidates proved ineligible upon scrutiny of their methods, so the evidence base has not in fact grown since the parent review—a finding in itself. Second, reappraising the primary data surfaced a transcription error in the parent synthesis; although it did not alter any conclusion, it illustrates the value of returning to source counts. It is worth stating explicitly what this review adds and what it does not. The principal clinical findings—that early oxytocin shortens labour by about two hours without reducing operative delivery, and that hyperstimulation with foetal heart rate changes may be more frequent—were already reported in the parent Cochrane review [6], and we do not present them as new. The contribution of the present work lies elsewhere: in the restriction to a clinically homogeneous nulliparous population in spontaneous labour with confirmed slow progress; in an updated search demonstrating that the evidence base has genuinely not expanded; in de novo extraction and recalculation from the primary reports; in updated RoB 2 and GRADE assessment supported by a sensitivity analysis of the variance estimator; and in the identification and correction of a transcription error carried in the previous synthesis. To these we now add a third observation of the same kind. A report that meets the eligibility criteria on its face, published in 2011 and not indexed in any database we searched, could not be included because the confidence intervals it reports are not reproducible from the sample size it states, and the discrepancy cannot be resolved from the published text [22]. Reviews of small evidence bases are particularly exposed to problems of this kind, since a single unverifiable dataset can dominate a pooled estimate; checking that reported intervals are arithmetically consistent with the stated denominators is an inexpensive safeguard and one we would encourage. We also note that this report reached us only because a peer reviewer drew it to our attention.

The one positive finding—hyperstimulation—deserves caution rather than emphasis. The pooled estimate is dominated by a single trial, its confidence interval only marginally excludes unity, events are sparse, and the two contributing trials did not define the outcome identically: in one, the “with foetal heart rate changes” category was a single case of hyperstimulation with persistent late decelerations, whereas in the larger trial the reported hyperstimulation count was not explicitly qualified by foetal heart rate change. Under the Hartung–Knapp sensitivity analysis, the interval no longer excluded unity. Taken together with the very low GRADE rating, the finding is best characterised as a possible safety signal rather than an established effect: enough to warrant vigilance when oxytocin is started early, but not enough to support a causal claim, and we have worded the Abstract, Results and Conclusions accordingly. The exact specification of the Hinshaw outcome cannot be recovered from the published report, and a definitive resolution would require access to the original trial documentation.

The strengths of this review are its clinically coherent population, duplicate extraction verified against primary sources with an internal-consistency check, de novo recomputation, and transparent handling of a definitional mismatch in postpartum haemorrhage. The limitations are inherited from the trials. All three were open-label, and the decision to perform caesarean or instrumental delivery is clinician-driven and therefore susceptible to ascertainment bias whose direction cannot be known. The trials were individually and collectively underpowered for caesarean delivery. The delayed arm was heterogeneous in both the length of delay and the proportion ultimately treated, which dilutes the contrast the review sets out to measure and inflates heterogeneity for the time-to-delivery and instrumental-delivery outcomes. This is not merely a statistical source of heterogeneity; it alters the clinical meaning of the comparison itself. What the pooled estimate describes is early augmentation versus a heterogeneous set of expectant or deferred-augmentation strategies, not a contrast between two standardised oxytocin-timing protocols, and the shorthand “early versus delayed oxytocin” should be read with that qualification throughout. A further limitation is statistical: with two or three trials per outcome, the between-study variance cannot be estimated reliably, so the DerSimonian–Laird intervals reported as primary are likely to be too narrow, as the Hartung–Knapp sensitivity analysis illustrates. With so few trials, we accept that narrative interpretation of the individual results carries at least as much weight as any pooled figure, and we have presented the trial-level data alongside every pooled estimate so that readers can make that judgement. Finally, although the seed set from the parent review was updated by an independent search of MEDLINE, Embase and CENTRAL with citation tracking, CINAHL and Web of Science were not searched independently; a trial indexed only in those sources could in principle have been missed, and the conclusions should be read in that light. A related and more general limitation is that a report carrying no DOI and published in a journal indexed by none of the databases searched is invisible to any search strategy of this kind, however carefully executed; such reports can surface only by chance or, as here, by referral. We cannot exclude the existence of others.

For practice, these data do not demonstrate a clear maternal or neonatal benefit from routine early augmentation: earlier initiation offers a shorter labour but no reduction in operative delivery and a possible increase in hyperstimulation. That is a different statement from demonstrating that delayed augmentation is superior, which this evidence cannot support. The recommendation of contemporary guidelines to confirm delay before augmenting [2,4] rests on broader clinical reasoning rather than on the three trials analysed here; our findings are consistent with it but do not by themselves establish it, and they are better used to inform individualised decision-making than to prescribe a preferred timing protocol. How slow progress is confirmed is itself part of the problem: across all three trials, the diagnosis rested on digital vaginal examination, which is subjective and poorly reproducible for both cervical dilatation and foetal head position, and this imprecision—compounded by the recognition that normal labour is slower and more variable than the classic curves assumed [3]—is one source of the clinician-driven ascertainment bias noted above. Objective intrapartum ultrasound assessment of foetal head position and station, as set out in the WAPM–PMF clinical practice guideline, could sharpen the distinction between true dysfunctional labour and foetal malposition and help target augmentation to the women most likely to benefit while sparing those who are in fact progressing normally [23]; sonographic assessment of foetal head flexion and position in the protracted active phase has been associated with labour outcome and may reveal the mechanical component of dystocia that oxytocin cannot address [24,25]. Supportive intrapartum care forms part of the same picture: effective analgesia influences maternal comfort, mobility and the perception of labour duration, and therefore the clinical decisions taken during a slow labour. Walking epidural techniques using ropivacaine or bupivacaine combined with fentanyl have been reported to provide comparable analgesia with high maternal satisfaction and without adverse obstetric or neonatal outcomes [26] and can be used alongside any oxytocin-timing strategy; the epidural rates in the included trials, which ranged from 53% to 97%, are a reminder that augmentation decisions are taken in this supportive context rather than in isolation. For research, the outstanding need is a blinded or otherwise bias-resistant trial, adequately powered for caesarean delivery, using a uniform definition of the delayed comparator and of clinically significant hyperstimulation. Until such a trial exists, the role of early oxytocin in the first labour remains, as it has for four decades, a treatment in search of the benefit for which it is given.

## 5. Conclusions

Among nulliparous women with spontaneous dysfunctional labour, early rather than delayed oxytocin may shorten labour by approximately two hours. Its effects on caesarean and instrumental delivery and on clinically important maternal and neonatal outcomes remain uncertain: the confidence intervals exclude neither benefit nor harm, and the absence of a significant difference does not establish equivalence. A possible increase in uterine hyperstimulation was observed, but the two contributing trials did not define the outcome identically, the estimate depends almost entirely on one trial, and it did not survive sensitivity analysis; it should be regarded as an uncertain signal. Certainty was low for most outcomes and very low for hyperstimulation. These findings concern when to begin oxytocin once slow progress has been diagnosed, and do not by themselves establish either that routine early augmentation is beneficial or that delayed augmentation is superior. Until an adequately powered, bias-resistant trial with a uniform comparator and a uniform hyperstimulation definition is available, the timing of augmentation is best individualised.

## Figures and Tables

**Table 1:** Characteristics of the included trials.

	Bidgood 1987 [9]	Dencker 2009 [10]	Hinshaw 2008 [11]
Country	UK	Sweden	UK (12 units)
Randomised (early/delayed)	40/20	314/316	208/204
Diagnosis of slow progress	<0.5 cm/h	Arrest ≥ 2 h or <1 cm/3 h	≤2 cm over 4 h from 3 to 6 cm
Membranes	Ruptured before randomisation	Amniotomy first if intact	Amniotomy in all if intact
Early arm	Immediate (low- or high-dose)	Within 20 min	Within intended 20 min
Delayed arm	Deferred 8 h	Withheld 3 h	Withheld 8 h
Delayed arm actually treated	5/20 after 8 h	87% (13% none)	30/204 within 8 h
Blinding	Open	Open	Open
Epidural rate	97%	72% vs. 76%	53% vs. 59%
Notes	60 of planned 150 recruited; two oxytocin arms combined	37 randomised in error; ITT retained	Stopped at 412 of planned 584 (funding)

**Table 2:** Risk of bias (RoB 2) by domain.

Trial	Randomisation	Deviations	Missing Data	Measurement	Selective Reporting	Overall
Bidgood 1987 [9]	Some concerns	Some concerns	Some concerns	High (open, clinician-driven)	Some concerns	High
Dencker 2009 [10]	Low	Some concerns (porous delayed arm)	Low	Some concerns	Some concerns	Some concerns
Hinshaw 2008 [11]	Low	Low	Low	Some concerns	Some concerns	Some concerns

**Table 3:** Summary of findings (GRADE).

Outcome	Trials (Women)	Effect (95% CI)	I^2^	Certainty
Caesarean delivery	3 (1102)	RR 0.86 (0.63–1.16)	0%	⊕⊕◯◯
Uterine hyperstimulation (trial-specific definitions)	2 (472)	RR 2.51 (1.04–6.06)	0%	⊕◯◯◯
Instrumental vaginal delivery	3 (1102)	RR 1.03 (0.66–1.61)	69%	⊕⊕◯◯
Spontaneous vaginal delivery	3 (1102)	RR 1.04 (0.88–1.25)	59%	⊕⊕◯◯
Apgar < 7 at 5 min	3 (1102)	RR 1.02 (0.43–2.40)	0%	⊕⊕◯◯
NICU admission	2 (1042)	RR 1.03 (0.64–1.66)	0%	⊕⊕◯◯
Postpartum haemorrhage > 1000 mL	1 (630)	RR 0.58 (0.28–1.20)	—	⊕⊕◯◯
Postpartum haemorrhage > 500 mL	1 (412)	RR 0.89 (0.61–1.30)	—	⊕⊕◯◯
Time to delivery (hours)	2 (1042)	MD −2.17 (−3.54 to −0.80)	90%	⊕⊕◯◯

RR, risk ratio; MD, mean difference; CI, confidence interval; FHR, foetal heart rate; NICU, neonatal intensive care unit. RR < 1 favours early oxytocin except for hyperstimulation, where RR > 1 indicates more events with early oxytocin. Sensitivity analysis (REML with Hartung–Knapp adjustment): caesarean delivery RR 0.86 (95% CI 0.56–1.32); hyperstimulation RR 2.51 (95% CI 0.41–15.36) ⊕⊕◯◯ low. ⊕◯◯◯ Very low.

## Data Availability

All data analysed in this review are contained within the article, are available in the primary trial reports cited, and are provided in full in the Supplementary Material Table S1 (data extraction workbook, which contains the per-arm data used for every analysis together with the verification log). Analytic code is available from the corresponding author upon request.

## Supplementary Materials

The following supporting information can be downloaded at https://www.mdpi.com/article/10.3390/healthcare14183000/s1. Table S1: Data extraction workbook (study ledger, characteristics, per-arm outcome data, pooled results, verification log). Table S2: Search execution pack (Database search strategies, search log and PRISMA counts). Table S3: Completed PRISMA 2020 checklist. Table S4: Reports excluded at full text, with reasons. Table S5: Outcome-specific risk-of-bias judgements (RoB 2), with rationales.
