# Supplementary material for: Early Versus Delayed Oxytocin Augmentation in Nulliparous Women with Spontaneous Dysfunctional Labour: A Systematic Review and Meta-Analysis
Source: Healthcare (Basel). 2026 Sep 14;14(18):3000. doi: 10.3390/healthcare14183000 (PMC13606038; doi:10.3390/healthcare14183000)
Supplement: Supplementary file 1 [file healthcare-14-03000-s001.zip › Supplementary_Tables_S2_S5.docx]

**Table S2. Search execution pack**

Early versus Delayed Oxytocin Augmentation in Nulliparous Women with Spontaneous Dysfunctional Labor: A Systematic Review and Meta-Analysis

The search strings below reproduce the strategy as executed and must be checked against the search history exported from each database before submission; the record counts in Table S2b are those reported in Section 3.1 and Figure 1.

**S2a. Database search strategies**

| **Database (platform)** | **Line** | **Search string** |
| --- | --- | --- |
| MEDLINE (PubMed) Inception to 16 July 2026 No language or publication-status limits | #1 | "Oxytocin"[Mesh] OR oxytocin[tiab] OR syntocinon[tiab] OR pitocin[tiab] |
|  | #2 | "Labor, Obstetric"[Mesh] OR "Dystocia"[Mesh] OR labour[tiab] OR labor[tiab] OR dystocia[tiab] OR "slow progress"[tiab] OR "failure to progress"[tiab] OR "prolonged labour"[tiab] OR "prolonged labor"[tiab] |
|  | #3 | augment*[tiab] OR stimulat*[tiab] OR "early"[tiab] OR "delayed"[tiab] OR timing[tiab] OR expectant[tiab] |
|  | #4 | nullipar*[tiab] OR primipar*[tiab] OR primigravid*[tiab] OR "first labour"[tiab] OR "first labor"[tiab] |
|  | #5 | randomized controlled trial[pt] OR controlled clinical trial[pt] OR randomi*[tiab] OR trial[tiab] |
|  | #6 | #1 AND #2 AND #3 AND #4 AND #5 |
| Embase (Elsevier) Inception to 16 July 2026 | #1 | 'oxytocin'/exp OR oxytocin:ti,ab OR syntocinon:ti,ab |
|  | #2 | 'labor'/exp OR 'dystocia'/exp OR labo?r:ti,ab OR dystocia:ti,ab OR 'slow progress':ti,ab OR 'prolonged labo?r':ti,ab |
|  | #3 | augment*:ti,ab OR stimulat*:ti,ab OR early:ti,ab OR delayed:ti,ab OR timing:ti,ab OR expectant:ti,ab |
|  | #4 | nullipar*:ti,ab OR primipar*:ti,ab OR primigravid*:ti,ab |
|  | #5 | 'randomized controlled trial'/exp OR randomi*:ti,ab OR trial:ti,ab |
|  | #6 | #1 AND #2 AND #3 AND #4 AND #5 |
| CENTRAL (Cochrane Library) Inception to 16 July 2026 Trials only | #1 | MeSH descriptor: [Oxytocin] explode all trees OR (oxytocin OR syntocinon):ti,ab,kw |
|  | #2 | MeSH descriptor: [Labor, Obstetric] explode all trees OR MeSH descriptor: [Dystocia] explode all trees OR (labour OR labor OR dystocia OR "slow progress" OR "prolonged labour"):ti,ab,kw |
|  | #3 | (augment* OR stimulat* OR early OR delayed OR timing OR expectant):ti,ab,kw |
|  | #4 | (nullipar* OR primipar* OR primigravid*):ti,ab,kw |
|  | #5 | #1 AND #2 AND #3 AND #4 |

**S2b. Search log and PRISMA counts**

| **Stage** | **n** | **Notes** |
| --- | --- | --- |
| MEDLINE/PubMed | 255 | Search executed 16 July 2026 |
| Embase | 580 | Search executed 16 July 2026 |
| CENTRAL | 244 | Search executed 16 July 2026 |
| Total from database searching | 1079 |  |
| Identified by other methods | 1 | Awan 2011, referred by a peer reviewer; no DOI and not indexed in any database searched, therefore not retrievable by the strategy above |
| Duplicates removed | 446 | Embase–MEDLINE overlap 202; CENTRAL 244 |
| Unique records screened | 633 |  |
| Excluded at title/abstract | 626 |  |
| Full texts assessed | 8 | 7 from database searching; 1 from reviewer referral |
| Excluded at full text | 5 | See Table S4 |
| Studies included | 3 | Bidgood 1987; Dencker 2009; Hinshaw 2008 (1102 women) |

**Supplementary methods.** The trials included in Cochrane review CD007123 were used as the seed set and the search above was run to update it. Forward citation tracking of the three included trials was performed, and the reference lists of the included trials and of relevant guidelines were screened. CINAHL and Web of Science were not searched independently; this is stated as a limitation in the manuscript. No language or publication-status restriction was applied and no automation tool was used at any stage.

**Table S3. PRISMA 2020 checklist**

Locations refer to the revised manuscript. Items highlighted in yellow are those whose content changed during peer review (updated study selection, the post hoc sensitivity analyses, the withdrawal of the funnel plot, and the outcome-specific risk-of-bias and GRADE reporting).

| **Section and topic** | **Item #** | **Checklist item** | **Location where item is reported** |
| --- | --- | --- | --- |
| **TITLE** | | | |
| Title | 1 | Identify the report as a systematic review. | Title page — the title states “A Systematic Review and Meta-Analysis”. |
| **ABSTRACT** | | | |
| Abstract | 2 | See the PRISMA 2020 for Abstracts checklist. | Abstract (structured, 200 words). Reports objective, eligibility, sources, synthesis method, number of included studies, results with contributing trials and participants for each principal outcome, certainty and interpretation. |
| **INTRODUCTION** | | | |
| Rationale | 3 | Describe the rationale for the review in the context of existing knowledge. | Section 1, paragraphs 1–2 — divergence between active-management practice and WHO recommendations; limitations of existing syntheses that pool mixed populations. |
| Objectives | 4 | Provide an explicit statement of the objective(s) or question(s) the review addresses. | Section 1, final paragraph — whether the timing of oxytocin alters mode of delivery, intrapartum safety or duration of labor in nulliparous women. |
| **METHODS** | | | |
| Eligibility criteria | 5 | Specify the inclusion and exclusion criteria for the review and how studies were grouped for the syntheses. | Section 2.1 — population, intervention, comparator and design criteria; labour phase restricted to the established active first stage; trial-specific diagnostic thresholds retained rather than a single common criterion. |
| Information sources | 6 | Specify all databases, registers, websites, organisations, reference lists and other sources searched or consulted. Specify the date when each source was last searched. | Section 2.2 — Cochrane review CD007123 as seed set; MEDLINE (via PubMed), Embase and CENTRAL from inception to 16 July 2026; forward citation tracking and reference-list screening. One further report was identified through peer-reviewer referral (Section 3.1, Figure 1). CINAHL and Web of Science were not searched independently. |
| Search strategy | 7 | Present the full search strategies for all databases, registers and websites, including any filters and limits used. | Section 2.2; full search strings, date limits, filters and search log in Table S2. |
| Selection process | 8 | Specify the methods used to decide whether a study met the inclusion criteria, including how many reviewers screened each record, whether they worked independently, and any automation tools used. | Section 2.3 — two reviewers screened and extracted independently; disagreements resolved by a third reviewer. No automation tools were used. |
| Data collection process | 9 | Specify the methods used to collect data from reports, including how many reviewers collected data, whether they worked independently, any processes for obtaining or confirming data from investigators, and any automation tools used. | Section 2.3 — duplicate extraction onto a piloted form; per-arm counts taken from the primary reports; internal-consistency check that modes of delivery summed to each randomised denominator; all estimates recomputed de novo. |
| Data items | 10a | List and define all outcomes for which data were sought. Specify whether all results compatible with each outcome domain were sought, and if not, the methods used to decide which results to collect. | Sections 2.3 and 3.3–3.6 — caesarean delivery (primary); instrumental and spontaneous vaginal delivery; uterine hyperstimulation as defined in each trial; 5-min Apgar < 7; neonatal intensive care admission; postpartum haemorrhage by each trial’s own definition; time from randomisation to delivery. |
| Data items | 10b | List and define all other variables for which data were sought. | Table 1 — country, randomised numbers, diagnostic criterion for slow progress, membrane status, timing in each arm, proportion of the delayed arm actually treated, blinding, epidural rate, trial-specific notes. |
| Study risk of bias assessment | 11 | Specify the methods used to assess risk of bias in the included studies, including details of the tool(s) used, how many reviewers assessed each study and whether they worked independently. | Section 2.4 — Cochrane RoB 2, per outcome, independently by two reviewers. Trial-level summary in Table 2 and Figure 2; outcome-specific judgements with rationales in Table S5. |
| Effect measures | 12 | Specify for each outcome the effect measure(s) used in the synthesis or presentation of results. | Section 2.5 — risk ratios with 95% confidence intervals for dichotomous outcomes; mean differences for continuous outcomes. |
| Synthesis methods | 13a | Describe the processes used to decide which studies were eligible for each synthesis. | Sections 2.1 and 3.1 — trials eligible for the review contributed to each synthesis for which they reported the outcome; contributing trials are named for every pooled estimate. |
| Synthesis methods | 13b | Describe any methods required to prepare the data for presentation or synthesis. | Section 2.5 — continuity correction of 0.5 applied only to cells with zero events; postpartum haemorrhage analysed by each trial’s definition rather than pooled across dissimilar thresholds. |
| Synthesis methods | 13c | Describe any methods used to tabulate or visually display results of individual studies and syntheses. | Figure 2 (RoB 2 traffic-light plot); Figures 3–4 (forest plots with per-trial counts and weights); Tables 1–3. |
| Synthesis methods | 13d | Describe any methods used to synthesise results and provide a rationale for the choice(s). If meta-analysis was performed, describe the model(s), method(s) to identify the presence and extent of statistical heterogeneity, and software package(s) used. | Section 2.5 — DerSimonian–Laird random-effects model prespecified because the duration of delay and the criteria for initiating oxytocin in the control arm were not uniform; heterogeneity quantified by I² and τ², with the caveat that I² estimated from two or three trials is not evidence of homogeneity. StatsDirect, with sensitivity analyses in R (metafor). |
| Synthesis methods | 13e | Describe any methods used to explore possible causes of heterogeneity among study results. | Sections 2.5, 3.8 and 4 — no subgroup or meta-regression analysis was possible with three trials; heterogeneity was explored narratively (non-uniform delayed comparator, non-equivalent outcome definitions) and by leave-one-out analysis. |
| Synthesis methods | 13f | Describe any sensitivity analyses conducted to assess robustness of the synthesised results. | Sections 2.5 and 3.8 — post hoc sensitivity analyses: τ² re-estimated by REML with the Hartung–Knapp confidence-interval adjustment; fixed-effect (inverse-variance and Mantel–Haenszel) estimates; leave-one-out analysis for both dichotomous outcomes. |
| Reporting bias assessment | 14 | Describe any methods used to assess risk of bias due to missing results in a synthesis (arising from reporting biases). | Section 2.5 — funnel plot and Egger’s test were prespecified for outcomes informed by ten or more trials only; no outcome met this threshold, so no formal assessment was performed and no funnel plot is presented. |
| Certainty assessment | 15 | Describe any methods used to assess certainty (or confidence) in the body of evidence for an outcome. | Section 2.4 — GRADE; for the primary outcome, imprecision judged against an optimal information size. |
| **RESULTS** | | | |
| Study selection | 16a | Describe the results of the search and selection process, from the number of records identified in the search to the number of studies included in the review, ideally using a flow diagram. | Section 3.1 and Figure 1 — 1079 records identified by database searching and 1 by peer-reviewer referral; 633 unique records screened; 8 full texts assessed; 3 trials (1102 women) included. |
| Study selection | 16b | Cite studies that might appear to meet the inclusion criteria, but which were excluded, and explain why they were excluded. | Section 3.1 and Table S4 — five full-text exclusions with study-specific reasons: Blanch 1998 and Hemminki 1985 (mixed parity, not separable); Mohd Faizal 2022 (slow progress not confirmed, induced labours not separable); Mohamed 2022 (randomised variable was dose, not timing); Awan 2011 (reported confidence intervals not reproducible from the stated sample size, discrepancy unresolvable from the published report). |
| Study characteristics | 17 | Cite each included study and present its characteristics. | Section 3.2 and Table 1 — Bidgood 1987, Dencker 2009, Hinshaw 2008. |
| Risk of bias in studies | 18 | Present assessments of risk of bias for each included study. | Section 3.2, Table 2 and Figure 2 (trial-level summary by domain, as a traffic-light plot) and Table S5 (outcome-specific judgements with rationales). |
| Results of individual studies | 19 | For all outcomes, present, for each study, summary statistics for each group and an effect estimate and its precision, ideally using structured tables or plots. | Figures 3–4 present per-arm counts, per-trial risk ratios with 95% confidence intervals and weights; Sections 3.3–3.6 give per-trial data in the text for the remaining outcomes. |
| Results of syntheses | 20a | For each synthesis, briefly summarise the characteristics and risk of bias among contributing studies. | Sections 3.2–3.6 — contributing trials named for each synthesis, with the risk-of-bias context stated in Section 3.2. |
| Results of syntheses | 20b | Present results of all statistical syntheses conducted, including summary estimates and their precision and measures of statistical heterogeneity. | Sections 3.3–3.6 and Table 3. |
| Results of syntheses | 20c | Present results of all investigations of possible causes of heterogeneity among study results. | Sections 3.8 and 4 — leave-one-out results and narrative discussion of the non-uniform comparator and non-equivalent outcome definitions. |
| Results of syntheses | 20d | Present results of all sensitivity analyses conducted to assess the robustness of the synthesised results. | Section 3.8 and Table 3 footnote — REML with Hartung–Knapp: caesarean delivery RR 0.86 (95% CI 0.56–1.32), hyperstimulation RR 2.51 (95% CI 0.41–15.36); fixed-effect estimates; leave-one-out results for both outcomes. |
| Reporting biases | 21 | Present assessments of risk of bias due to missing results (arising from reporting biases) for each synthesis assessed. | Section 2.5 — not assessed; no outcome was informed by the prespecified minimum of ten trials. Not downgraded for publication bias in GRADE (Section 3.7). |
| Certainty of evidence | 22 | Present assessments of certainty (or confidence) in the body of evidence for each outcome assessed. | Section 3.7 and Table 3 — GRADE ratings with the domains downgraded and the reason for each downgrade stated per outcome. |
| **DISCUSSION** | | | |
| Discussion | 23a | Provide a general interpretation of the results in the context of other evidence. | Section 4, paragraphs 1–3 — including an explicit statement of what this review adds beyond the parent Cochrane review and what it does not claim as new. |
| Discussion | 23b | Discuss any limitations of the evidence included in the review. | Section 4, paragraph 5 — open-label design and decision bias; underpowering; clinical heterogeneity of the delayed comparator; instability of the variance estimate with two or three trials. |
| Discussion | 23c | Discuss any limitations of the review processes used. | Section 4, paragraph 5 — CINAHL and Web of Science not searched independently; reports carrying no DOI and published in journals indexed by none of the databases searched cannot be reached by the search strategy, and one such report was identified only through peer-reviewer referral. |
| Discussion | 23d | Discuss implications of the results for practice, policy and future research. | Section 4, final paragraph, and Section 5 — findings distinguished from guideline-derived recommendations; research priority is an adequately powered, bias-resistant trial with a uniform comparator and a uniform hyperstimulation definition. |
| **OTHER INFORMATION** | | | |
| Registration and protocol | 24a | Provide registration information for the review, including register name and registration number, or state that the review was not registered. | Section 2 — PROSPERO, registration number CRD420261451210. |
| Registration and protocol | 24b | Indicate where the review protocol can be accessed, or state that a protocol was not prepared. | Section 2 — protocol prepared per PRISMA-P and accessible through the PROSPERO record. [**Authors to confirm the public URL.**] |
| Registration and protocol | 24c | Describe and explain any amendments to information provided at registration or in the protocol. | Sections 2.5 and 3.8 — three analyses were added after registration, in response to peer review: REML with the Hartung–Knapp adjustment, fixed-effect estimation and leave-one-out analysis. All are reported as post hoc. No prespecified analysis was removed; the prespecified reporting-bias plan was applied as written, and a funnel plot supplied with the original submission was withdrawn because it was inconsistent with that plan. |
| Support | 25 | Describe sources of financial or non-financial support for the review, and the role of the funders or sponsors in the review. | Funding statement — no external funding was received. |
| Competing interests | 26 | Declare any competing interests of review authors. | Conflicts of Interest statement — none declared. |
| Availability of data, code and other materials | 27 | Report which of the following are publicly available and where they can be found: template data collection forms; data extracted from included studies; data used for all analyses; analytic code; any other materials used in the review. | Data Availability Statement and Supplementary Materials — Table S1 (data extraction workbook, including the per-arm data used for all analyses and the verification log), Table S2 (search execution pack), Table S3 (this checklist), Table S4 (full-text exclusions), Table S5 (outcome-specific RoB 2). Analytic code is available from the corresponding author on request. |

Adapted from: Page, M.J.; McKenzie, J.E.; Bossuyt, P.M.; et al. The PRISMA 2020 statement: an updated guideline for reporting systematic reviews. *BMJ* **2021**, *372*, n71.

**Table S4. Reports excluded at full text, with reasons**

| **Study** | **Citation** | **Eligibility criterion not met** | **Reason for exclusion** |
| --- | --- | --- | --- |
| Blanch 1998 | Blanch, G.; Lavender, T.; Walkinshaw, S.; Alfirevic, Z. Dysfunctional labour: A randomised trial. Br. J. Obstet. Gynaecol. 1998, 105, 117–120. | Population: nulliparous women only | Enrolled nulliparous and parous women in a single randomised cohort; nulliparous outcome data could not be separated from the published report. |
| Hemminki 1985 | Hemminki, E.; Lenck, M.; Saarikoski, S.; Henriksson, L. Ambulation versus oxytocin in protracted labour: A pilot study. Eur. J. Obstet. Gynecol. Reprod. Biol. 1985, 20, 199–208. | Population: nulliparous women only | Reported outcomes for a mixed-parity sample without a nulliparous subgroup. |
| Mohd Faizal 2022 | Upawi, S.N.; Ahmad, M.F.; Abu, M.A.; Ahmad, S. Amniotomy and early oxytocin infusion vs amniotomy and delayed oxytocin infusion for labour augmentation amongst nulliparous women at term: A randomised controlled trial. Midwifery 2022, 105, 103238. | Population: confirmed slow progress; spontaneous labour | Women were enrolled at 4 cm dilatation rather than after a diagnosis of slow progress, and 16.5% of labours were induced with no separable outcome data. Belongs to the literature on early amniotomy with oxytocin. |
| Mohamed 2022 | Mohamed, S.A.; Abbas, A.M.; Mostafa, E.G.A. High dose versus low dose oxytocin for augmentation of delayed labour in obese women: A randomized clinical trial. Egypt. J. Hosp. Med. 2022, 87, 1953–1960. | Intervention: timing of oxytocin as the randomised variable; nulliparous population | The randomised variable was oxytocin dose (high versus low), not timing, in an obese parous population. Belongs to the high-versus-low-dose literature. |
| Awan 2011 | Awan, A.S.; Bakhtiar, U.; Amin, G. Outcome of early versus delayed oxytocin augmentation in nulliparous women on the duration of labour and other obstetric and neonatal outcome — a randomized controlled trial. J. Islam. Int. Med. Coll. 2011, 6, 14–19. (No DOI; not indexed in MEDLINE, Embase or CENTRAL.) | Data must be verifiable against the sample described | Meets the eligibility criteria on its face. The reported confidence intervals are not reproducible from the stated sample size: for the arm sizes given (157/158), the interval around the reported caesarean odds ratio of 0.8 would be approximately 0.39–1.71 rather than the 0.5–1.4 reported, and around the reported instrumental-delivery odds ratio of 1.5 approximately 0.81–2.86 rather than 0.97–2.4; the reported intervals correspond to a sample of roughly twice the stated size. No per-arm event counts are given from which the discrepancy could be resolved. Excluded because the reported estimates could not be verified against the sample described. |

Identified through peer-reviewer referral: Awan 2011. All other reports were identified by the database search. No study was excluded on grounds of language or publication status.

**Table S5. Outcome-specific risk-of-bias judgements (RoB 2), with rationales**

These judgements reproduce and extend Table 2 and Figure 2 of the manuscript. They are the assessments of the two independent reviewers and must be confirmed against the assessment forms before submission.

D1, randomisation process; D2, deviations from intended interventions; D3, missing outcome data; D4, measurement of the outcome; D5, selection of the reported result.

| **Outcome** | **Trial** | **D1** | **D2** | **D3** | **D4** | **D5** | **Overall** | **Rationale** |
| --- | --- | --- | --- | --- | --- | --- | --- | --- |
| Caesarean delivery; instrumental vaginal delivery; spontaneous vaginal delivery | Bidgood 1987 | S | S | S | H | S | High | Open label. The decision to proceed to operative delivery is taken by the attending clinician, so knowledge of allocation could act on the outcome itself and not only on its ascertainment. 60 of a planned 150 women recruited; two oxytocin arms combined. |
|  | Dencker 2009 | L | S | L | S | S | Some concerns | Open label, with the same decision-bias concern. 13% of the delayed arm never received oxytocin and a further ~20% received it earlier than allocated; ITT retained. |
|  | Hinshaw 2008 | L | L | L | S | S | Some concerns | Open label, with the same decision-bias concern. 30 of 204 women allocated to delay received oxytocin within 8 hours. Trial stopped at 412 of a planned 584 for funding reasons. |
| Uterine hyperstimulation (trial-specific definitions) | Bidgood 1987 | S | S | S | H | S | High | Outcome neither blinded nor defined identically across trials: here explicitly qualified by fetal heart rate change (one event, with persistent late decelerations). |
|  | Hinshaw 2008 | L | L | L | H | S | High | Hyperstimulation reported without stating that every event was accompanied by fetal heart rate abnormality. Detection is dependent on unblinded intrapartum monitoring and on the threshold for intervention. |
| Apgar < 7 at 5 min; NICU admission | Bidgood 1987 | S | S | S | L | S | Some concerns | Objectively recorded outcomes, unlikely to be influenced by knowledge of allocation; the measurement domain is therefore rated low despite the open design. |
|  | Dencker 2009 | L | S | L | L | S | Some concerns | As above. |
|  | Hinshaw 2008 | L | L | L | L | S | Some concerns | As above. NICU admission reported. |
| Postpartum haemorrhage | Dencker 2009 | L | S | L | S | S | Some concerns | Threshold >1000 mL. Estimated blood loss is measured without blinding and is observer dependent. A transcription error in the parent review (42 rather than the 45 reported) was identified and corrected. |
|  | Hinshaw 2008 | L | L | L | S | S | Some concerns | Threshold >500 mL; not pooled with the above because the definitions differ. |
| Time from randomisation to delivery | Dencker 2009 | L | S | L | L | S | Some concerns | Objectively timed. Diluted by the porous delayed arm rather than by measurement error. |
|  | Hinshaw 2008 | L | L | L | L | S | Some concerns | As above. |

L, low risk; S, some concerns; H, high risk.
